# Prominent protumoral cellular compartments of the tumor microenvironment in triple-negative breast cancer

**DOI:** 10.3389/fcell.2025.1668583

**Published:** 2026-01-08

**Authors:** Shuai Sun, Pan Zhao, Changrong Wang, Junjun Du, Tingting Zhang, Xiangyun He, Zhibo Zuo, Nan Li, Rongjing Zhou

**Affiliations:** 1 Department of Pathology, Hangzhou Cancer Hospital, Hangzhou, China; 2 Pathology Residency Training Base, Hangzhou First People’s Hospital Affiliated with Westlake University, Hangzhou, China; 3 Department of Pathology, Westlake University Affiliated Hangzhou First People’s Hospital, Hangzhou, China; 4 Department of Pathology, the Second Affiliated Hospital of Anhui Medical University, Hefei, China

**Keywords:** chemotherapy, drug resistance, immunotherapy, triple-negative breast cancer, tumor microenvironment

## Abstract

Triple-negative breast cancer (TNBC) is a highly aggressive subtype of breast cancer characterized by the absence of estrogen, progesterone, and HER2 receptor expression. This malignancy is often associated with a poor prognosis, early recurrence, and limited treatment options. The tumor microenvironment (TME) in TNBC plays a pivotal role in tumor progression, immune evasion, and therapeutic resistance. In recent years, an increasing body of evidence has highlighted the critical interactions between cancer cells and the components within the TME, including immune cells and soluble components. These interactions influence not only the biological behavior of the tumor but also its response to treatment. Exploring the complex interplay between tumor cells and immune components continues to inform the development of more effective therapeutic approaches. In this study, we provide a synopsis of advancements regarding the TME in TNBC. In light of different cellular compartments, we delineate multiscale interplays within the stroma–tumor symbiosis and highlight their antitumor functions and promising targeting strategies.

## 1 Introduction

Triple negative breast cancer (TNBC) is characterized by a lack of the expression of progesterone and estrogen receptors and overexpression or amplification of human epidermal growth factor receptor 2 (HER2). TNBC represents approximately 12%–18% of all breast cancers and is susceptible to visceral metastasis, prone to recurrence, and heterogeneous in nature (Foulkes et al., 2010). TNBC presents a dismal 5-year overall survival of 66% and 13% for local and metastatic diseases, respectively (Li et al., 2025). Even in patients with early-stage disease cured with surgical resection and systemic treatment, a high risk of recurrence and ensuing poor outcome threatens most TNBC patients (Lee, 2023). Despite the unifying defining term, TNBC is inherently heterogeneous both between and within patients (Wang X. et al., 2024). Genomic and transcriptomic profiling have categorized TNBC into different subtypes, which is instructive in personalizing treatment. One notable example is a classifier that divides TNBC into four groups: basal-like (BL1 and BL2), mesenchymal (M), and luminal androgen receptor (LAR) subtypes (Lehmann et al., 2016). High mutation level and ensuing immunogenicity are well-documented hallmarks of TNBC (Loizides and Constantinidou, 2023). For example, advanced sequencing analyses have shown that TNBC typically harbors a higher number of somatic mutations, a high frequency of TP53 mutations, and increased aneuploidy (Gao et al., 2016; Yates et al., 2015). Recently, researchers have focused on the heterogeneity not only inside tumor parenchyma but also within the TME in TNBC.

**FIGURE 1:**
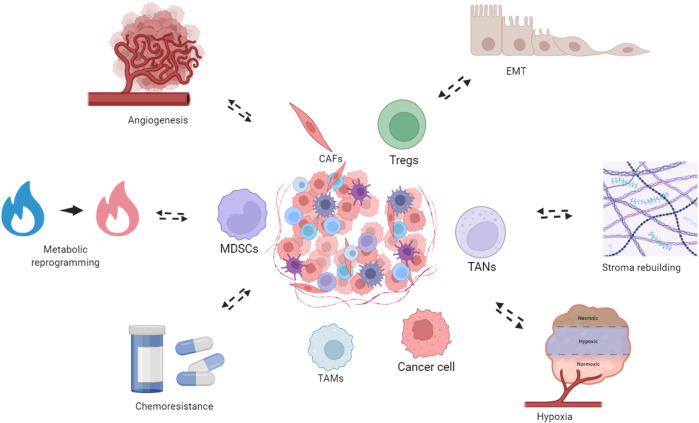
Bidirectional interactions between the TNBC TME and tumor-associated biological phenomena. The TNBC TME constitutes the structural foundation underlying multiple tumor biological phenomena, which in turn dynamically remodel the TME. These processes include metabolism reprogramming, where cancer cells and stromal components undergo metabolic adaptations to meet the bioenergetic demands of rapid proliferation. Concurrently, the TME promotes angiogenesis, facilitating the formation of new vasculature to supply oxygen and nutrients to the expanding tumor mass. The TME also drives epithelial-to-mesenchymal transition (EMT), enabling cancer cells to acquire enhanced migratory and invasive capabilities. Additionally, stroma remodeling occurs through extracellular matrix reorganization and recruitment of cancer-associated fibroblasts, creating a supportive niche for tumor progression. Hypoxic conditions within the TME further exacerbate tumor aggressiveness and contribute to therapeutic resistance. Importantly, cellular and metabolic components of the TME actively mediate chemoresistance, limiting the efficacy of cytotoxic agents. These processes also reciprocally modulate the TME. Created in https://BioRender.com.

The TME is composed of various acellular and cellular compartments, which together orchestrate various tumor behaviors such as angiogenesis and chemoresistance (Figure 1) (Wang et al., 2020). It has long been known that stromal cells are indispensable in endorsing cancer hallmarks through vigorous cellular communication and cell–stroma contact (Pietras and Östman, 2010). Three forms of intimate cell–cell communication exist between stroma and tumor parenchyma, i.e., soluble factors, cellular adhesion, and small extracellular vesicles (Ohyashiki et al., 2018). Immunomodulatory functions are embedded in many, if not most, of the cellular compartments in the TME (Hinshaw and Shevde, 2019). The TME can be considered part of the whole immune system, which is hijacked by the cancer, leading to a deviation from homeostasis that sustains immune evasion (Demaria et al., 2010).

New therapeutic options have improved the therapeutic armamentarium for TNBC (Cortes et al., 2022). Nevertheless, the current standards of care for TNBC patients remain far from satisfactory. In addition, current therapeutic options have been established more empirically, with the underlying mechanisms warranting further detailed elucidation (Yang et al., 2022). Exploring the TME may provide new rationales as the tumor and surrounding stroma function as a holistic unit, and current options lack a comprehensive understanding in this regard. Current standard therapies for TNBC, including chemotherapy and newly incorporated immunotherapy, inherently depend on modulating the tumor immune microenvironment (Li et al., 2023). In addition, advances in single-cell RNA sequencing and spatial transcriptional techniques have enabled unprecedented resolution in studies concerning cellular compartments in the TME (Tanay and Regev, 2017). In this review, we explored recent advances concerning the TME in TNBC in light of several major protumoral cellular compartments. We discussed the primary malignant biological behaviors of TNBC and their TME underpinnings. Finally, the reciprocal effects between treatment modalities—especially novel options and TME—are also incorporated.

## 2 Cancer-associated fibroblasts

**FIGURE 2:**
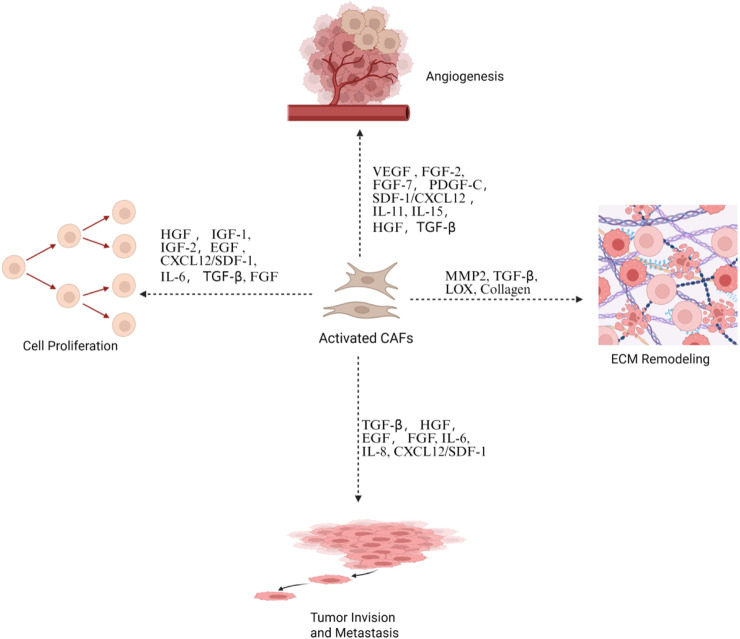
Activated CAFs promote tumor progression. This is through the secretion of growth factors and cytokines that drive angiogenesis, cell proliferation, and ECM remodeling and facilitate tumor invasion and metastasis. Created in https://BioRender.com.

**FIGURE 3:**
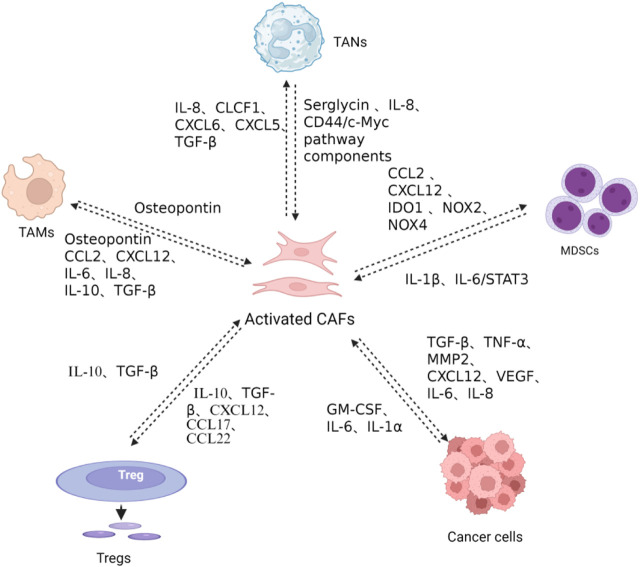
CAF-mediated immunosuppressive networks in the TME. CAFs recruit monocytes and promote M2-polarized TAM differentiation *via* secretion of osteopontin (OPN), CCL2, CXCL12, IL-6, IL-8, and transforming growth factor-β (TGF-β). CAFs facilitate regulatory T cell (Treg) recruitment and differentiation through the secretion of serglycin, IL-8, CCL2, Myc pathway components, CCL2, CXCL12, IDO1, NOX2, NOX4, IL-10, TGF-β, and CCL22, while Tregs reciprocally activate CAFs *via* IL-10 and TGF-β secretion, inducing collagen and extracellular matrix production that promotes tumor invasion and metastasis. CAFs recruit tumor-associated neutrophils (TANs) through IL-8, CLCF1, CXCL6, and TGF-β secretion, promoting N2 polarization, while TANs reciprocally induce CAF polarization toward an inflammatory phenotype through IL-1β secretion and promote CAF-tumor cell crosstalk *via* the IL-6/STAT3 pathway. CAFs recruit and polarize myeloid-derived suppressor cells (MDSCs) *via* the CCL2/CCR2 axis and CXCL12 secretion, with CAF-induced MDSCs generating immunosuppressive ROS through IDO1, NOX2, and NOX4 to suppress CD8^+^ T cell proliferation and IFN-γ production. MDSCs reciprocally support CAF activation through IL-1β secretion and IL-6/STAT3 pathway activation. CAFs directly promote tumor cell proliferation, invasion, and angiogenesis through secretion of TGF-β, TNF-α, MMP2, CXCL12, VEGF, IL-6, and IL-8. Conversely, cancer cell-derived IL-1α, IL-6, and GM-CSF stimulate CAF activation and cytokine production. Created in https://BioRender.com.

Adjacent fibroblasts are implicated in an array of human malignancies and are referred to as cancer-associated fibroblasts (CAFs). CAFs constitute a major contributor to the acellular components of the TME by producing extracellular matrix (ECM) proteins such as collagen, fibronectin, and laminin and secreting various growth factors, cytokines, and matrix metalloproteinases (MMPs). In addition, CAFs also serve as a primary producer of soluble factors in the TME. Intuitively, CAFs enclose tumor parenchyma and restrict its outgrowth, thereby exerting a physical antitumor effect. In addition, it has been suggested that co-culturing with fibroblasts suppresses the invasive abilities of breast cancer cells (Hu et al., 2008). Despite these antitumor facets, accumulating knowledge has unveiled a more multifaceted and largely protumoral role of CAFs in tumor development (Figure 2) (Zhang et al., 2013). In general, CAFs are associated with tumor invasion, progression, and drug resistance in breast cancer (Costa et al., 2014). A potential prerequisite for tumor metastasis is the presence of topographic factors that guide disseminated tumor cells. Baschieri et al. (2023) reported that CAFs deposit a tubular network in the tumor periphery, guiding subsequent cancer cells during colon cancer development. These membranous tubules generated by CAFs are designated as migrasomes, which can also directly enter surrounding cells to deliver cytokines from CAFs (Jiang et al., 2025). CAFs are known to produce an array of cytokines and mediate cellular communication (Figure 3). Stromal cell-derived factor-1 (SDF-1) is a well-established molecule effector of CAFs that is implicated in many human malignancies (Roccaro et al., 2014). An early *in vivo* study showed that CAF-deprived SDF-1 promotes the proliferation of breast cancer cells (Allinen et al., 2004). In line with this, Orimo et al. (2005) demonstrated that a C-X-C chemokine receptor 4 (CXCR4) antibody abrogates cellular proliferation, suggesting a pivotal role for the SDF-1/CXCR4 axis in this context. In addition to SDF-1, αSMA is another marker of activated fibroblasts (i.e., myofibroblasts), which predicts high-grade tumors and recurrence of breast cancer (Benyahia et al., 2017; Toullec et al., 2010). Additionally, fibroblast activation protein (FAP) is another surface marker of CAFs, the depletion of which leads to acute post-ischemia cancer hypoxic necrosis through the prothrombotic effects of IFN-γ and tumor necrosis factor- α (TNF-α) in Lewis lung carcinoma 2 expressing ovalbumin (LL2/OVA) tumors (Kraman et al., 2010).

Accumulating evidence has demonstrated the protumoral role of CAFs; however, strategies targeting CAFs do not necessarily produce the expected effects. For example, unselected elimination of nearby CAFs led to paradoxically unfavorable outcomes in pancreatic ductal adenocarcinoma (PDAC) due to the underlying role of CAFs (Ozdemir et al., 2014). Hence, subtyping CAFs and identifying the antitumor subgroup in TNBC may prevent otherwise ineffective therapeutic attempts. Initial functional distinctions have led to the coining of myofibroblastic CAFs and inflammatory CAFs, which represent two ends of the CAF spectrum (Biffi and Tuveson, 2021). Recently, distinct functional subsets of CAFs have been characterized in TNBC. In the research by A. Costa et al. (2018), four subgroups of fibroblasts in tumor and non-tumor breast tissue were delineated, with the CAF-S1 and CAF-S4 subsets comprising the majority of CAFs in TNBC. CAFs were sorted according to the expression levels of CD29, FAP, αSMA, platelet-derived growth factor receptor β (PDGFRβ), fibroblast-specific protein 1 (FSP1), and caveolin-1 (CAV1). A prominent CAF-S1 subset corresponds to the reduction of CD8^+^ T-cell content and the high number of FOXP3^+^ T cells in the TNBC tumor bed, which play an immunosuppressive role and are a potential intervention target (A. Costa et al., 2018). Similarly, Fan et al. (2024) revealed a group of tetraspanin 8^+^ myofibroblastic CAFs in breast cancer that underlie chemoresistance. Chemotherapy remains the mainstay of TNBC treatment, although resistance impairs the efficacy. Targeting specific functional subgroups of stromal cells may offer new avenues to circumvent chemoresistance. Given the complexity of the CAF-centric regulatory network and the overlapping or even opposing roles of a specific signaling pathway under different contexts, the prospect of a one-size-fit-all small molecule inhibitor or antibody targeting CAFs seems remote. However, the underlying signaling pathways and functional subtyping warrant further investigation to elucidate the role of CAFs in TNBC and develop novel treatment options. CAFs are also involved in the intimate mutual interaction between cellular compartments in the TME. For example, the aforementioned study showed that the modulatory effect of CAF-S1 on CD4^+^ CD25^+^ T cells is mediated through junctional adhesion molecule 2 (JAM2) in CAFs and OX40 ligand (OX40L) and PD-L2 in the T cells. In addition to T cells, CAFs are also implicated in interplay with cancer stem cells (CSCs) in TNBC (A. Costa et al., 2018a). CSCs play a major role in TNBC chemoresistance (Lytle et al., 2018). CSCs comprise a functionally defined subgroup of tumor cells with well-established implications in tumorigenesis and chemoresistance across malignancies (Oskarsson et al., 2014). The exact gene-expression definition and surface markers of CSCs remain to be fully elucidated, although several molecules, such as CD133 and CD166 and components of the Wnt signaling pathway, have been proposed (O Brien et al., 2007; Todaro et al., 2007). The TME regulates the CSC population through Wnt expression. Mechanistically, Wnt signaling modulates PD-L1 expression in CSCs in TNBC (Castagnoli et al., 2019). The TME also shelters CSCs by establishing protective niches, in which a subset of CD10^+^ GPR77^+^ CAFs play important roles (Vermeulen et al., 2010). The function of both normal stem cells and CSCs depends on the surrounding specialized niches (Korkaya et al., 2011). Novel notions, such as CSC-related niches, emphasize a focus on the spatial interactions of cells rather than on surface molecules. In nearby niches, surrounding cells, mainly CAFs, signal to CSCs to maintain their features and reverse differentiation through hepatocyte growth factor (HGF)/β-catenin signaling (Vermeulen et al., 2010). In a mouse model mimicking the aged-skin microenvironment, CAFs were reported to establish niches characterized by immunosuppression and inflammation, protecting incipient tumor cells from CD8^+^ T cells (Ruhland et al., 2016). Targeting specialized niches of CSCs and supporting CAFs may offer new avenues to circumvent resistance. Although CAFs have not emerged as a major target in TNBC treatment, the mechanisms regulating them are being elucidated. Given the abundance of CAFs in the TNBC TME, it is worthwhile to further elucidate the interplays between CAFs and other cell compartments.

Transforming growth factor-β (TGF-β) plays a critical and multifaceted role in shaping the immunosuppressive TME in TNBC. TGF-β plays paradoxical roles in cancer progression. It acts as a tumor suppressor in early stages but promotes tumor development and metastasis in advanced stages (Shi et al., 2022; Tang et al., 2003). The interplay between TGF-β signaling and CAFs plays a pivotal role in TNBC progression. CAF-regulated TGF-β ligands drive fibrosis accumulation, collagen deposition, and metastasis in the TNBC TME (Zheng et al., 2024). Conversely, TGF-β activation in CAFs induces their metabolic reprogramming, characterized by increased oxidative stress, autophagy, and glycolysis, along with downregulation of caveolin-1 (Guido et al., 2014). This metabolic shift transforms CAFs into a catabolic phenotype that generates nutrients such as L-lactate and chemical building blocks that fuel mitochondrial metabolism and support the anabolic growth of adjacent breast cancer cells. This is termed the reversed Warburg effect (Liang et al., 2022). In addition, TGF-β can also convert fibroblasts into CAFs through autophagy-dependent pathways (Huang et al., 2021). Extracellular matrix CAFs (ecmCAFs) have been identified as a fibroblast subpopulation with distinct ECM-associated characteristics that contribute to a fibrotic tumor microenvironment (Lu et al., 2025). These ecmCAFs play a critical role in creating physical barriers that exclude immune cells from the tumor. In this regard, TGF-βs suppress the activity of matrix-degrading proteases through the induction of protease inhibitors such as plasminogen activator inhibitor 1 and tissue inhibitors of metalloproteinases. Beyond ECM remodeling, α-SMA^+^ CAFs have been identified as an important cellular source of TGF-β, which hampers the cytotoxic activity of CD8^+^ T cells (Zheng and Hao, 2024).

## 3 Tumor-associated neutrophils

In addition to prominent roles in acute inflammation, neutrophils have also been implicated in the development of malignancies (Coffelt et al., 2016). High-level circulating neutrophils predict unfavorable prognosis in malignancies (Adler et al., 2023; Szczerba et al., 2019). In addition, tumor-associated neutrophils (TANs) are also implicated in several aspects of antitumor immunity, including immune cell recruitment and antigen presentation (Linde et al., 2023; Pylaeva et al., 2022). High-level neutrophil-to-lymphocyte ratio (NLR) also predicts unfavorable prognosis in breast cancer patients (Guo et al., 2019). A meta-analysis involving 2,355 patients with TNBC showed that higher NLR correlates with both unfavorable DFS and OS (Liu K. et al., 2022). In agreement with this, Wang M. et al. (2024) reported that neutrophils are associated with lymph node metastases in breast cancer. Historically, the short half-life and low cellular mRNA content of TANs have limited studies on these cells. Recently, however, advances in single-cell RNA sequencing (scRNA-seq) approaches have revealed the versatile role of TANs (Summers et al., 2010; Wigerblad et al., 2022). For example, Wu et al. (2024) characterized distinct TAN subgroups with respect to less-known aspects, including angiogenesis and antigen presentation, through single-cell RNA sequencing. Swarming TANs, tumor cells and other TME cells interact closely in a spatially coordinated manner. Using physically interacting cell RNA-sequencing (PIC-seq), Camargo et al. (2025) reported that TANs and tumor cells are physically interacting cells (PICs) in breast cancer, suggesting a close functional dependency. Typically, neutrophils function through phagocytosis, degranulation, and extrusion of neutrophil extracellular traps (NETs) (Silvestre-Roig et al., 2019). In an infection state, NETs trap microorganisms, whereas in malignancy, they co-opt disseminated tumor cells and facilitate metastasis in the form of tumor–neutrophil symbiosis (Szczerba et al., 2019). In addition to binding and sheltering tumor cells, the DNA component of NETs is implicated in cancer metastasis as DNAse treatment inhibits the pro-metastatic effects of NETs (Tohme et al., 2016). Chemotherapy features collective cellular death and tissue impairment. Neutrophils typically act as first responders in this local stress state. In breast cancer lung metastasis models, Mousset et al. (2023) demonstrated that integrin αvβ1 locates in NETs and activates TGF-β to facilitate epithelial-to-mesenchymal transition (EMT) and ensuing chemoresistance. Similarly, a report showed that NETs promote gastric cancer malignant behaviors through TGF-β signaling and that the inhibitor LY 2157299 disrupts cancer metastasis (Xia et al., 2022).

## 4 Tumor-associated macrophages

Macrophages play dual roles in tumor progression across different malignancies (Nasir et al., 2023). Overall, pro-inflammatory antitumor M1 and anti-inflammatory M2 type macrophages demonstrate two extreme polarization states. However, this dichotomy is largely a conceptual simplification of tumor-associated macrophages (TAMs), between which exists a continuum of intermediate states (Wang et al., 2023). In liver cancer, anti-inflammatory M2 phenotypes were induced by IL-4, IL-10, and IL-13, which promote tumor stroma remodeling and create an immunosuppressive microenvironment, thereby boosting malignancy (Capece et al., 2013; Ju and Tacke, 2016). In addition, TAMs release CCL22 and epidermal growth factor (EGF) to recruit regulatory T cells (Tregs) and build an immunosuppressive microenvironment (Kitamura et al., 2015). Tumor growth requires the formation of new vascular structures to meet its nutritional and oxygen demands. Targeting tumor-related angiogenesis represents one therapeutically viable avenue in oncology. However, inhibitors of angiogenesis such as bevacizumab alone or together with chemotherapy failed to provide sufficient benefit in TNBC patients (Kümler et al., 2014; Symonds et al., 2019). It might be attributed to the complexity involved in the regulation of angiogenesis, and finding new options is of clinical relevance. TAMs play a well-documented role in angiogenesis and vasculogenesis. In breast cancers, approximately 50% of neovascularization is repressed after macrophage depletion. Ensuing hypoxia leads to necrosis in tumor areas, providing a rationale for designing antitumor treatments that target TAM-mediated angiogenesis. However, such methods should be applied cautiously and fine-tuned to avoid otherwise unfavorable overall outcomes. Similar to other cellular compartments, TAMs are also involved in intimate interplays in the cell–cell communication network of the TME. It was reported that the switch between M1 and M2 polarization involves T-lymphocyte modulation in the breast cancer mouse mammary tumor virus–polyomavirus middle T antigen (MMTV-PyMT) model. Taken together, as the role of macrophages in TNBC continues to unfold, targeting immunosuppressive TAMs in combination therapies represents a promising avenue that warrants further investigation.

## 5 Myeloid-derived suppressor cells

Myeloid-derived suppressor cells (MDSCs) are a group of key immunosuppressive immune cells with CD11b and Gr1 expression (Holmgaard et al., 2015). In tumorigenesis, chronic inflammation is a well-documented risk factor. The tumor-initiating effect of inflammation is partially attributed to the induction of MDSCs, which represent a major repressor of T cells and NK cells. Given their profound immunosuppressive effects, MDSCs have been metabolically likened to the queen bee for tumors in evading immune surveillance (Tesi, 2019). In ontogeny, MDSCs are similar to immature neutrophils and monocytes and are classified into two main subsets: monocytic myeloid-derived suppressor cells (M-MDSCs) and polymorphonuclear myeloid-derived suppressor cells (PMN-MDSCs). Despite controversies over the intrinsic nature of MDSCs, they play distinct regulatory roles in pathologic conditions. In breast cancer lung metastasis 2 (LM2) orthotopic xenograft model, cancer cell-derived CXCL1/2 was shown to increase S100A8/9 expression in MDSCs, which in turn enhances breast cancer cell viability under chemotherapy. Furthermore, CXCL1/2 is also elevated in LM2 metastatic cells compared with parental MDA-MB-231 cells, and knockdown of CXCL1/2 led to reduced metastasis (Acharyya et al., 2012). Subsequently, recruited MDSCs produce immunosuppressive components such as reactive oxygen species (ROS) and arginase 1 to suppress CD8^+^ T cells and NK cells in breast cancer pre-metastatic niches (PMNs) (Wang et al., 2019). In addition to the local TME, MDSCs also appear in the blood, bone marrow, and spleen. All of them are powered with similar suppressor attributes. In breast cancer, MDSCs are also accountable for the low and short response to immunotherapy. Given its noticeable role in suppressing antitumor immunity, modulating MDSCs represents a promising route in cancer treatment. In a drug repurposing study, Mabrouk et al. (2023) showed that glyceryl trinitrate, together with doxorubicin, suppressed PMN-MDSCs through reduced Stat5 phosphorylation to reduce tumor growth in a mouse model of TNBC.

## 6 T cells

Depending on the quantity and location of infiltrating T cells, solid tumors can be categorized into the following phenotypes: immune-excluded, immune-desert, and immune-inflamed (Gerard et al., 2021). Immune-inflamed tumors are characterized by abundant T-cell infiltration within the tumor parenchyma. Immune-excluded tumors have T cells restricted to the tumor margins or stroma without penetrating the tumor nests. Immune-desert tumors exhibit minimal T-cell infiltration throughout both the tumor core and surrounding stroma. T cells comprise a major type of immune cells in the TME, among which CD8^+^ cytotoxic T cells are the main effector cells in antitumor immunity. Immune escape is closely associated with T-cell exhaustion and suppression. Suppression of T cell-mediated cytotoxicity is observed in many, if not most, TMEs. The presence of CD8^+^ T cells is indicative of a better prognosis and therapeutic response in many malignancies, including breast cancer (Fridman et al., 2012; Ali et al., 2014). In TNBC, it was estimated that approximately 60% of tumors hold CD8^+^ T-cell infiltration (Stanton et al., 2016). Another pooled analysis, which enrolled 2,148 patients from 9 studies, demonstrated that early-stage TNBC patients with pronounced tumor-infiltrating lymphocytes (TILs) correlate with favorable outcomes. Notably, a cohort study showed that pT1C TNBC patients with more than 75% of stromal TILs had 98% 10-year breast cancer-specific survival even in the absence of neoadjuvant or adjuvant chemotherapy (Geurts et al., 2024). For patients with less than 30% stromal TILs in the stage I cohort, the 10-year breast cancer-specific survival decreased to 87%. The favorable survival outcomes observed in patients with high T-cell infiltration, even without chemotherapy, suggest the prominent role of T lymphocytes in mediating antitumor immunity. In summary, CD8^+^ T-cell infiltration yields a favorable outcome in early-stage TNBC when an otherwise immunosuppressive TME is not fully established. With advances in the characterization of fine-grained cell types, different subsets of CD8^+^ T cells in the TME have been identified. In Byrne et al. (2020), a subgroup of CD8^+^ T cells, namely, tissue-resident memory (TRM) T cells, was characterized and found to predict better prognosis and treatment response in early-stage and metastatic TNBC.

During progression, cancers develop various mechanisms to evade T-cell cytotoxicity. One of these mechanisms is the downregulation of antigen presentation (Patel et al., 2017). In pancreatic cancer, the survival of disseminated cancer cells was attributed to reduced expression of major histocompatibility complex class I (MHCI) protein (Pommier et al., 2018). Intravasation into the tumor is a necessary step for T-cell-mediated antitumor cytotoxicity as T cells are primarily primed in draining lymph nodes by antigen-presenting cells. In addition to the cytotoxic effect, T cells also serve as the pillar of the immunomodulatory network in the TME. DeNardo et al. (2009) reported that CD4^+^ T cells modulate the TAM phenotype and bioactivity through IL-4 signaling. IL-4 plays a central role as it induces EGF transcription of TAMs, subsequently promoting their polarization toward the M2 phenotype. Other cytokines implicated in this setting include IL-13 and IL-10, as opposed to IFN-γ and IL-17, which promote an M1 phenotype. The state of the TME is shaped by vigorous interplays between chemokine ligands and receptors, which are inherently promiscuous (DeNardo et al., 2009). Targeting the chemokine network represents a promising avenue in remodeling the TNBC TME. Chen et al. (2020) showed that the intratumoral administration of CCL25 increased the number of CCR9^+^ CD8^+^ T cells, which are more readily activated in antitumor immune responses. Notably, the drug delivery nanoparticle system in this study is sensitive to acidity in the tumor TME and thus decreases the possible off-target effect (Chen et al., 2020). Given the pivotal role of T cells in tumor immunity, it is not surprising that their activities are corrupted in nearly all TMEs. Much of the therapeutic failure and immune escape can be attributed to this fact, with T-cell exhaustion playing a major role. The success of immune checkpoint inhibitors has exploited mechanisms to unleash the tumoricidal potential of CD8^+^ T cells by blocking co-inhibitory receptors. Potential future routes of therapeutic strategies include redirecting CD8^+^ T-cell localization, enhancing CD8^+^ T-cell function, and relieving immunosuppression.

In addition to directly enhancing cytotoxicity, TME modulation strategies also involve targeting negative cellular regulation. Typically, the presence of Tregs expressing the transcription factor forkhead box protein P3 (FOXP3) predicts an unfavorable prognosis across malignancies. Notably, the discovery and characterization of Tregs earned the 2025 Nobel Prize in Physiology or Medicine, underscoring their fundamental importance in immune regulation. Infiltrates of Tregs are associated with unfavorable survival in breast cancer (Bates et al., 2006; Merlo et al., 2009). Mechanistically, Tregs express inhibitory molecules, including cytotoxic T-lymphocyte-associated protein 4 (CTLA-4), inducible T-cell costimulator (ICOS), glucocorticoid-induced TNFR-related protein (GITR), and OX40, both in tumors and tumor-draining lymph nodes. An increased Treg/NK cell ratio is observed in lymph nodes with breast cancer metastasis, wherein Tregs suppress the tumoricidal effect of NK cells (Kos et al., 2022). Notably, the immunosuppressive function of Tregs was neutralized in the pro-inflammatory TME, suggesting the context-dependent regulatory modes of Tregs (Overacre-Delgoffe et al., 2017).

## 7 Interplays among cellular compartments and the shared signaling pathways

The TNBC microenvironment is an integrated ecosystem rather than several isolated cellular compartments. The cellular interactions in the TME are primarily mediated through several key kinase signaling cascades. Sheng et al. (2023) showed that tumor-derived granulocyte colony-stimulating factor (G-CSF) extends the lifespan and increases their pro-metastatic functions *via* the phosphatidylinositol 3-kinase (PI3K) pathway. Meanwhile, TAN-derived relaxin-2 increases MCF7 cell migration *via* PI3K signaling (Sheng et al., 2023). Aberrations in the PI3K–AKT pathway are commonly observed in TNBC, with PIK3CA mutations detected in approximately 16% of cases (Martínez-Sáez et al., 2020). Blocking the mutated PI3K–AKT pathway has stimulated extensive research in breast cancer. The clinical results remain modest, which may be attributed to the complex compensatory mechanisms within the TME (R. L. B. Costa et al., 2018). Transcription factor Stat3 also serves as a critical hub mediating intercellular communication in the TNBC microenvironment. IL-6 is a key paracrine factor secreted by CAFs (Nagasaki et al., 2014). IL-6 activates Stat3 signaling in tumor cells, promoting stemness and chemoresistance (Korkaya et al., 2012). Reciprocally, tumor-derived factors [e.g., IL-6 and ROS] activate Stat3 in CAFs, reinforcing their myofibroblastic phenotype and ECM remodeling capacity. In addition, Stat3 induces expression of IL-23 through direct transcriptional activation of the *IL-23* gene in TAMs. Stat3 also inhibits NF-κB-dependent IL-12 gene expression in dendritic cells (Kortylewski et al., 2009). Mechanistically, Stat3 activation in Tregs enhances their suppressive function through Th17 responses (Chaudhry et al., 2009). This multi-directional Stat3 signaling exemplifies how a single pathway integrates signals across diverse cellular compartments to drive tumor-promoting effects.

NF-κB also lies at the hub of TME signaling. TAM-derived TNF-α and IL-1β activate NF-κB in tumor cells, driving inflammatory gene expression and promoting the EMT (Wu and Zhou, 2010). Simultaneously, tumor-derived exosomes containing oncogenic factors activate NF-κB in stromal cells, reprogramming them toward pro-tumorigenic phenotypes, such as CAFs (Paggetti et al., 2015). NF-κB also upregulates PD-L1 expression on both tumor cells and tumor-infiltrating myeloid cells through direct transcriptional activation and other mechanisms. These actions link cellular stress and inflammatory responses to immune evasion mechanisms (Antonangeli et al., 2020). While TGF-β exhibits tumor-suppressive effects in early-stage disease, advanced TNBC often hijacks this pathway for immune suppression and metastasis promotion (Bhola et al., 2013; Hao et al., 2019). CAFs secrete TGF-β and other immunosuppressive factors that promote the differentiation and activation of Tregs (Kinoshita et al., 2013). TGF-β also directly suppresses CD8^+^ T-cell proliferation and function, creating an immunosuppressive niche that excludes these cytotoxic lymphocytes (Thomas and Massagué, 2005). TAMs represent another major source of TGF-β in the TME, with M2-polarized TAMs secreting substantial amounts of this cytokine to reinforce immunosuppression (Chen et al., 2019). TGF-β activation in MDSCs enhances their immunosuppressive capacity and facilitates their accumulation at tumor sites. They then further inhibit T-cell responses through multiple mechanisms, including arginase 1 and inducible nitric oxide synthase production (Ostrand-Rosenberg and Fenselau, 2018). Meanwhile, TGF-β acts on dendritic cells to impair their maturation and antigen-presenting capacity, thereby disrupting the priming of effective antitumor T cell responses (Kobie et al., 2003). In addition, TGF-β induces endothelial-to-mesenchymal transition in tumor endothelial cells, resulting in the downregulation of adhesion molecules such as vascular endothelial (VE)-cadherin and weakened endothelial barrier function (Ma et al., 2020).

## 8 Dual functionality of TME cellular components and implications for TNBC therapeutics

The nature of fibroblasts as a restraining barrier challenges simplistic therapeutic CAF depletion strategies (Brechbuhl et al., 2017). These cells, characterized by expression of α-SMA and type I collagen, form physical barriers that limit incipient breast cancer invasion. They also secrete secreted frizzled-related protein 2 (SFRP2) and Dickkopf-1 (DKK1) to antagonize Wnt signaling and suppress CSC properties (Bhattacharjee et al., 2021). Genetic ablation of αSMA+ CAFs in TNBC models accelerates tumor progression and reduces survival. In highly fibrotic pancreatic ductal adenocarcinoma (PDAC), a lower abundance of myofibroblasts in patients correlates with reduced survival. Additionally, depletion of myofibroblasts leads to increased immunosuppressive CD4^+^ FOXP3^+^ Tregs in mouse models (Ozdemir et al., 2014). These contribute to the complexity of targeting CAFs, even with their protumoral roles that we have discussed earlier. Subtyping CAFs and identifying the protumoral groups represent promising avenues. For example, single-cell RNA sequencing identifies at least four distinct CAF subtypes in breast cancer, each with unique functional profiles (A. Costa et al., 2018). Given the inter-patient heterogeneity of TNBC tumors, identifying conserved biomarkers of tumor-promoting CAF subpopulations warrants further investigation. It is essential for developing targeted therapies that can be personalized to individual CAF landscapes (Cao et al., 2025).

Beyond their well-established pro-tumorigenic functions, TANs also exhibit significant anti-cancer properties through the N1 polarization state. They mediate direct cytotoxicity *via* ROS production and activation of adaptive immune responses in breast cancer (Zhang et al., 2020). During early phases of tumor development, N1 TANs expressed high levels of TNF-α, ICAM-1, and Fas, promoting tumor cell apoptosis and enhancing CD8^+^ T-cell recruitment (Eruslanov et al., 2014; Obeagu and Ezeala, 2025). Early-stage lung cancer TME frequently shows N1 predominance, which stimulates and augments antitumor effector T-cell responses (Singhal et al., 2016). However, TGF-β exposure drives N2 polarization, characterized by arginase 1 expression, VEGF secretion, and immunosuppressive activity (Fridlender et al., 2009). The N1/N2 ratio is dynamic and reversible. IFN-β treatment or TGF-β blockade can reprogram N2 back to the N1 phenotype. Given that TNBC tumors secrete copious amounts of TGF-β and harbor abundant TANs, strategies targeting neutrophil phenotypic modulation hold clinical promise (SenGupta et al., 2021). Likewise, TAMs in breast cancer are predominantly M2-polarized with tumor-promoting functions. M1-like TAMs exert antitumor immunity through the secretion of pro-inflammatory cytokines such as IL-12, IL-6, IL-23, and TNF-α (Wigginton et al., 1996; Xie et al., 2016). Therefore, despite achieving substantial TAM depletion, CSF1R inhibition has minimal effects on antitumor immune responses in established tumors (O Brien et al., 2021). Under this context, strategies targeting TAM phenotypic switching warrant further investigation. Xu et al. (2015) showed that intratumoral delivery of IL-21 effectively reprograms TAMs from M2 to the M1 phenotype in breast cancer models. This phenotype switch rapidly stimulates T-cell responses and dramatically enhances therapeutic efficacy against HER2-positive tumors, suggesting its potential application in TNBC treatment (Xu et al., 2015).

MDSCs represent one of the most potent immunosuppressive populations in TNBC. MDSCs are traditionally characterized by their ability to suppress T-cell responses through multiple mechanisms. These include L-arginine depletion *via* arginase-1, nitric oxide production through inducible nitric oxide synthase (iNOS), and reactive oxygen species generation (Gabrilovich and Nagaraj, 2009). However, MDSCs presumably exist in an immature state that retains developmental plasticity. Under appropriate conditions, MDSCs can differentiate into immunostimulatory antigen-presenting cells rather than maintaining their suppressive phenotype (Condamine et al., 2016). For example, under inflammatory signaling, MDSCs can be induced to differentiate into mature dendritic cells and macrophages with antigen-presenting capacity (Kusmartsev and Gabrilovich, 2006). The MDSC suppressive function is not irreversibly fixed but rather maintained by specific factors. All-trans retinoic acid (ATRA) promotes MDSC differentiation into mature myeloid cells by inducing glutathione synthesis, which neutralizes the oxidative stress that maintains their immature state (Mirza et al., 2006). Similarly, vitamin D3 inhibits MDSC immunosuppressive function through VDR-mediated transcriptional changes, reducing arginase-1 expression and nitric oxide production (Fleet et al., 2020). Mechanistically, the suppression *versus* maturation fate decision hinges on the Stat3/Stat1 balance at least to some extent. Tumor-derived factors such as IL-6 and GM-CSF induce Stat3 activation, which maintains MDSC immaturity through sustained expression of S100A8/A9 proteins and prevents differentiation (Cheng et al., 2008). Likewise, Stat3 and Stat5 activation downregulates interferon regulatory factor 8 (IRF8), a transcription factor for myeloid differentiation, thereby promoting MDSC development over normal myeloid maturation (Cheng et al., 2008).

## 9 Metastasis

Metastasis accounts for over 90% of cancer-associated mortalities (Gupta and Massague, 2006). The term malignancy *per se* refers to neoplasms that possess the capacity to metastasize somewhat. Despite advances in treatment, metastatic tumors are hard to treat and largely incurable due to their systemic and highly therapy-resistant nature. Metastatic TNBC is a devastating diagnosis. The overall survival (OS) and progression-free survival (PFS) is 2–3 months and 5–8 months for pretreated metastatic TNBC patients, respectively (Kazmi et al., 2020). Furthermore, the overall 5-year survival rate is a dismal 13% (Bardia et al., 2024). Despite differences and advances, metastatic foci generally keep a similar microenvironment and mode of cell–cell contact with primary tumors (Chiang and Massagué, 2008). Metastatic loci develop from microscopic sites to manifest sites. Micrometastases [also referred to as pre-metastatic niches (PMNs)] appears before clinically overt metastases emerge. The development of metastatic sites requires not only the aggregation of tumor cells but also the establishment of a local protumoral ecosystem, which begins at the PMN stage. Previous studies have shown that a prerequisite for overt metastasis is the inducing effect of early-arrived bone marrow myeloid cells. Local and metastatic microenvironments are also implicated in other stages of metastasis. The invasion–metastasis cascade recapitulates the sequential steps of metastasis with the roles of TME embedded in every step. The basement membrane stands as the first frontier to be confronted with tumor cells, where CAFs play essential roles in the establishment of this specialized form of the ECM. Malignant cells invade either in a collective form or as single cells, involving participation of ECM-modulating components such as proteases and integrins. The ECM compartment is produced largely by CAFs. Its physical features, such as rigidity and thickness, partially dictate cancer invasiveness. Intravasation and extravasation are completed through interplays among disseminated tumor cells, endothelial cells, and pericytes (Fidler, 2003). Then, the arrival at specific organs necessitates cues from the secondary microenvironment. During these processes, most newly infiltrating cancer cells die due to the unfavorable milieu (Calon et al., 2012). It was noted that metastasis occurs not after complete outgrowth of primary lesions but rather emerges as early as when the primary lesion breaches the basal lamina (Oskarsson et al., 2014). This suggests that the heterogeneity between metastatic TME and primary TME results from tumor stochastic evolution. Luminal-like breast cancer holds a metastatic tropism toward bone, while TNBC tends to colonize viscera such as the brain and lung (Fulford et al., 2007). The moving tropism of exosomes partially accounts for this discrepancy. During the formation of lung PMNs, tumor-derived exosomes play an integral role in tumor–stroma communication. Hoshino et al. (2015) demonstrated a threefold increase of MDA-MB-231-derived exosomes in lungs compared with exosomes derived from other less lung-tropic cell lines. In addition, lung-tropic metastasis is also dependent on breast cancer cell-expressed chemokine receptors, such as CXCR4 and C-C receptor 7 (CCR7) (Müller et al., 2001). In addition to the lung, TNBC is also highly susceptible to brain metastasis. Approximately half of advanced TNBC patients would eventually manifest brain metastasis in the disease course (Balinda et al., 2024).

## 10 Hypoxia and metabolism reprogramming

In tumors, approximately 80% of glucose is consumed through aerobic glycolysis due to metabolic reprogramming, a phenomenon known as the Warburg effect (Gou et al., 2025). Enhanced aerobic glycolysis was initially observed in tumor cells but was later shown to be implicated in tumor-infiltrating immune cells. The reason underlying immune cells’ consumption of large amounts of energy lies in the energy-intensive nature of immune-related functions, including cytotoxicity and cytokine release (Buttgereit et al., 2000). Breast tumors are characterized by enhanced angiogenesis and vascularization. Energy-intensive processes are necessary to supply the growing tumor. It has been reported that endothelial cells produce 85% of their ATP through glycolysis (De Bock et al., 2013). Metabolic competition between tumor cells and immune cells dictates tumor progression to some extent. Lactic acid is the main byproduct of glycolysis. In many malignancies, high lactic acid concentration in the TME impairs mono-carboxylate transporter-1 (MCT-1) and leads to decreased proliferation and cytokine production of CTLs (Fischer et al., 2007). Furthermore, lactic acid, either independently or synergistically with immunosuppressive cytokines, can induce tumor-specific immature dendritic cells characterized by impaired antigen presentation function (Gottfried et al., 2006). In a mouse sarcoma model, reduced mechanistic target of rapamycin (mTOR) activity, glycolysis level, and IFN-γ production in T cells are attributed to tumor metabolism and are reversed by immune checkpoint blockade (Chang et al., 2015). This suggests the potential of targeting tumor metabolism to unleash antitumor immunity. In this context, sophisticated targeting strategies are required to prevent the suppression of antitumor T cells.

## 11 Therapy options for TNBC and their implications in the TME

**FIGURE 4:**
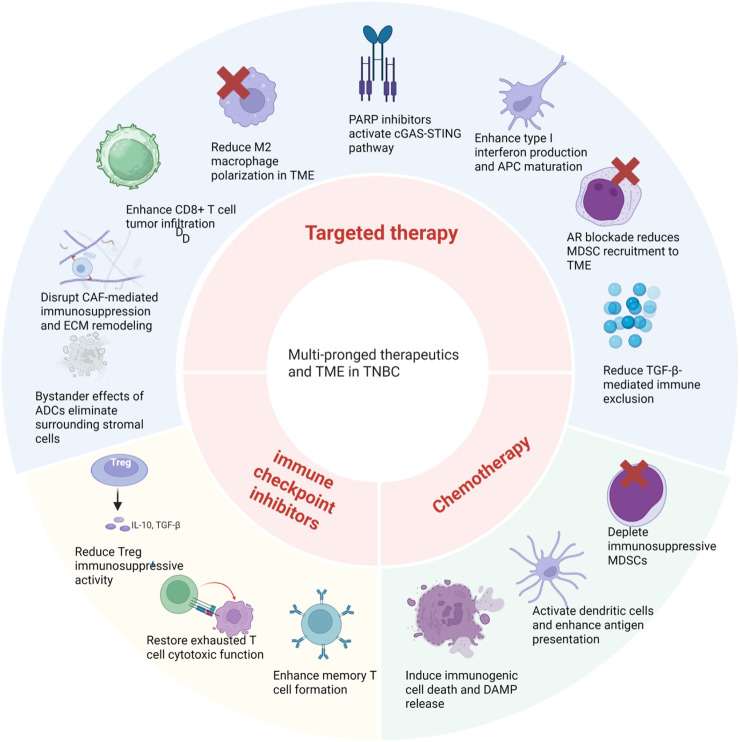
Multi-pronged therapeutics modulate the TME in different ways. Chemotherapy induces immunogenic cell death and DAMP release, activates dendritic cells to enhance antigen presentation, and depletes immunosuppressive MDSCs. Immune checkpoint inhibitors unleash cytotoxic T cells by blocking inhibitory molecules, restoring exhausted T cell function, enhancing memory T cell formation, and reducing Treg immunosuppressive activity. Targeted therapies exert pleiotropic effects on the TME. PARP inhibitors activate the cGAS–STING pathway to enhance type I interferon production and APC maturation. AR blockade reduces MDSC recruitment and TGF-β-mediated immune exclusion. ADC bystander effects eliminate surrounding stromal cells and disrupt CAF-mediated immunosuppression and ECM remodeling. Collectively, these multi-pronged therapeutic approaches reshape the immunosuppressive TME into an immune-permissive state, facilitating enhanced antitumor immunity in TNBC. Created in https://BioRender.com.

Currently, multimodal options are incorporated in TNBC treatment. In addition to directly eradicating tumor cells, these therapeutics also modulate the TME (Figure 4).

**FIGURE 5:**
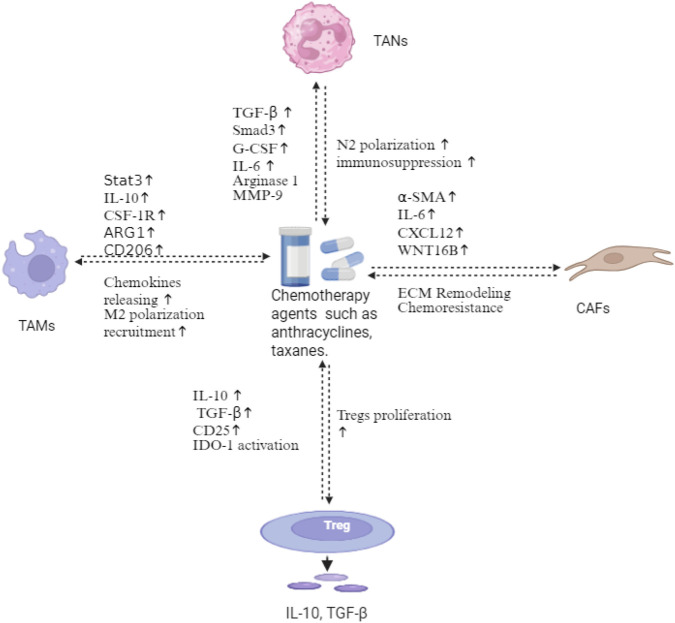
Chemotherapy-induced immunosuppressive remodeling in TNBC. Anthracyclines and taxanes trigger multifaceted alterations in the tumor microenvironment. TAMs undergo M2 polarization *via* Stat3, IL-10, CSF-1R, and ARG1 activation, releasing chemokines that amplify immunosuppression. TANs shift toward N2 polarization through TGF-β/Smad3, G-CSF, and IL-6 signaling, secreting α-SMA, IL-6, CXCL12, and Wnt16B. CAFs mediate ECM remodeling and chemoresistance. Chemotherapy also induces Treg proliferation through the upregulation of IL-10, TGF-β, CD25, and IDO-1, establishing an immunosuppressive feedback loop. This chemotherapy-induced network represents a key mechanism of therapeutic resistance in TNBC. Created in https://BioRender.com.

Chemotherapy remains the cornerstone in TNBC treatment, with its efficacy extending beyond direct cytotoxicity to complex remodeling of TME compartments (Figure 5). TNBC demonstrates remarkable sensitivity to anthracyclines and taxanes, achieving pathological complete response (pCR) rates of 22% *versus* 11% compared to non-TNBC in neoadjuvant settings (Liedtke et al., 2008). This enhanced chemosensitivity correlates with TNBC’s elevated mutational burden, contrasting with the generally lower mutational landscape in breast cancers (Bareche et al., 2018; Shah et al., 2012). Anthracyclines trigger immunogenic cell death (ICD), releasing damage-associated molecular patterns (DAMPs) that transform tumor cells into immunogenic entities, recruiting and activating CD8^+^ cytotoxic T lymphocytes (Kroemer et al., 2013). This process establishes a self-reinforcing cycle, leading to increased neoantigen release and T-cell priming. Chemotherapy also simultaneously depletes circulating lymphocytes and disrupts T-cell homeostasis. For example, cyclophosphamide causes selective depletion of Tregs at low doses while suppressing effector T cells at high doses (Ghiringhelli et al., 2007; Lutsiak et al., 2005). In patients with residual disease after neoadjuvant chemotherapy, the density and spatial distribution of tumor TILs strongly predict survival outcomes. Stromal TILs >30% correlate with improved disease-free survival (DFS) and OS. In addition, intratumoral CD8^+^ T cells demonstrate even stronger prognostic value (Denkert et al., 2018; Loi et al., 2019a). The KEYNOTE-522 trial revealed that patients achieving residual cancer burden-1 (RCB-1) scores attained more favorable event-free survival (EFS) compared to those with RCB-3 scores. Importantly, RCB-1 patients also demonstrated higher CD8^+^ T-cell infiltration than RCB-3 patients, suggesting that preserving T-cell function during chemotherapy improves outcomes (Schmid et al., 2020a). These observations underscore a critical paradigm in TNBC therapy. Although chemotherapy effectively induces tumor cell death and initial immune activation through ICD, its immunosuppressive effects may limit long-term effects. The correlation between preserved TIL function and improved outcomes in RCB-1 patients suggests that strategies to maintain or restore immune competence during chemotherapy may be beneficial. These may include optimal dosing regimens, sequential rather than concurrent immune checkpoint blockade, or selective lymphocyte protection methods. M2-polarized TAMs present as a major contributor of chemoresistance. Hughes et al. (2015) showed that chemotherapy triggers the accumulation of a specific M2-polarized TAM subpopulation (MRC1^+^ TIE2-hi CXCR4-hi) around blood vessels, where they promote tumor revascularization and relapse through VEGF-A release in breast cancer models. These perivascular TAMs are recruited *via* upregulated CXCL12/CXCR4 signaling in the post-chemotherapy TME (Hughes et al., 2015). These TAMs also secret IL-10, TGF-β, and VEGF, which collectively promote chemoresistance and metastatic dissemination in breast cancer (Munir et al., 2021). Pharmacologic blockade of CXCR4 could reduce M2 TAMs after chemotherapy, especially those close to blood vessels, reducing tumor vascularization and regrowth. Meanwhile, CXCL12/CXCR4 signaling promotes metastasis to lungs through the activation of PI3K/AKT in breast cancer (Luker and Luker, 2006). These suggest the potential multifaceted effects of targeting CXCL12/CXCR4 in breast cancer. In contrast, paclitaxel demonstrates unique TAM-modulating properties by promoting their reprogramming toward an M1-like phenotype through a TLR4-dependent mechanism (Wanderley et al., 2018). The reduction of M2 TAMs correlates with improved therapeutic responses as patients with low infiltration of CD163^+^ macrophages achieved a higher rate of pCR after neoadjuvant chemotherapy (Ye et al., 2021). Collectively, these findings suggest that chemotherapy may paradoxically enhance M2 TAM-mediated chemoresistance. Combining chemotherapy with TAM-targeting strategies, either blocking CXCR4 or promoting M1 polarization, could synergistically improve treatment outcomes and reduce the metastatic potential of TNBC.

**FIGURE 6:**
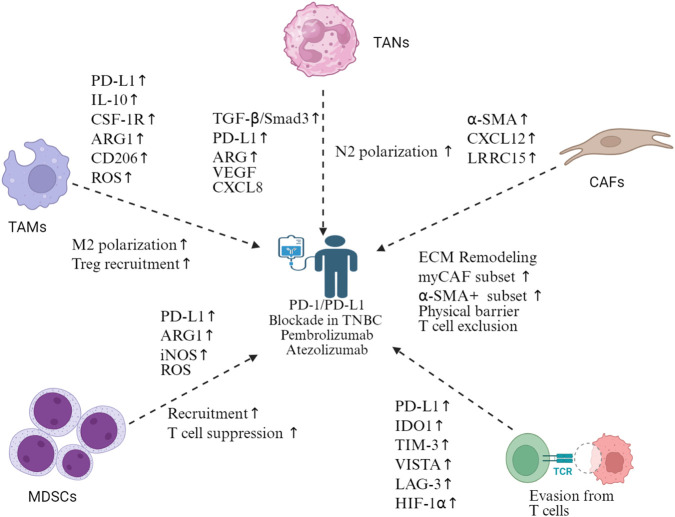
Mechanisms of immune checkpoint blockade resistance in TNBC. Multiple cellular compartments contribute to resistance against the PD-1/PD-L1 blockade in TNBC. TAMs promote M2 polarization and Treg recruitment *via* PD-L1, IL-10, CSF-1R, ARG1, CD206, and ROS upregulation. TANs undergo N2 polarization through TGF-β/Smad3, PD-L1, ARG, VEGF, and CXCL8 signaling. CAFs mediate ECM remodeling, expand myoCAF and α-SMA+ subsets, and create physical barriers causing T-cell exclusion *via* α-SMA, CXCL12, and LRRC15 expression. MDSCs suppress T-cell function through PD-L1, ARG1, iNOS, and ROS production. T-cell dysfunction manifests through the upregulation of exhaustion markers (PD-L1, IDO1, TIM-3, VISTA, and LAG-3) and HIF-1α, leading to T-cell evasion. This complex resistance network necessitates combination therapeutic approaches beyond single-agent immune checkpoint inhibition in TNBC. Created in https://BioRender.com.

Immune checkpoint inhibitors have brought a paradigm shift in cancer treatment. Despite encouraging results, cumulative approvals have been granted for an array of regimens in solid cancers. That said, only a relatively small proportion of patients benefit from immune checkpoint inhibitors. The TNBC subtype was shown to express higher PD-L1 level in cells of the TME among breast cancers (Ali et al., 2015). However, the TNBC TME may adapt quickly to immune checkpoint inhibitor therapies (Figure 6). In PD-L1-positive TNBC patients, the combination of nab-paclitaxel and atezolizumab significantly improved OS compared with single-agent chemotherapy (Schmid et al., 2020b). Overall, other breast cancer subtypes are relatively less immunogenic compared to TNBC. Even in the most immunogenic TNBC subtype, monotherapy immunotherapy benefits fewer than 20% of patients, not to mention the issue of acquired resistance (Adams et al., 2019a). Preliminary studies have established a correlation between TIL score and PD-1 therapy efficacy in TNBC, with patients having above-median TIL scores responding more favorably Loi et al., 2019b). The fifth edition of the WHO Classification of Breast Tumors has incorporated TIL evaluation for clinical guidance (Byrne et al., 2020). Likewise, the presence of PD-L1 is associated with a favorable outcome. A phase 1 study showed >1% TIL corresponded to a higher objective response rate (ORR) and longer OS in the enrolled 116 evaluable patients (Emens et al., 2019). Meanwhile, single-agent atezolizumab performed better in first-line metastatic TNBC. Furthermore, researchers in this field have also been innovating strategies to elicit better responses. In the phase 2 TONIC trial, 67 metastatic TNBC patients who received nivolumab were induced beforehand with one or none of the following: irradiation, cyclophosphamide, cisplatin, and doxorubicin (Voorwerk et al., 2019). The highest ORR was observed in patients induced with doxorubicin, with the response rate up to 35%, followed by cisplatin (23%). The following gene expression analysis demonstrated that two MDSC-related genes and two CD4 T-cell associated genes were upregulated after doxorubicin and cisplatin, while three myeloid cell-associated genes were enriched with the induction of nivolumab. Under this route, the incorporation of immunomodulatory agents into metastatic TNBC treatment warrants further investigation. Immunotherapies also include adoptive cell therapy and oncolytic virus therapy, *etc.* Adoptive cell therapy, represented by CAR-T cell therapy, showed an impressive effect in hematological malignancies. However, when it comes to breast cancer, the efficacy is largely unsatisfactory in preclinical models and early clinical trials, which is attributed to the immunosuppressive nature of the TME. Another field that has witnessed recent progress is oncolytic virus therapy. The basis of OVs in tumor immunity is similarly to that of chemotherapy, which arouse immunogenic cell death (Kepp et al., 2014; Pandha et al., 2016). In addition, corroborative evidence also showed OVs are implicated in immunomodulation, enhancing antigen presenting, all of which are associated with TME (Bourgeois-Daigneault et al., 2016; Zhang et al., 2014). Bourgeois-Daigneault et al. (2016) showed that Maraba-based OV not only improves outcomes when in combination with resection but also sensitizes response to immune checkpoint inhibitors in TNBC models. Furthermore, Maraba treatment showed lasting efficacy in the inoculation rechallenge model, which is attributed to virus replication. Despite these and similar reports, the field remains largely unexplored and requires more robust research, especially in combination with immune checkpoint inhibitors.

Approximately 57% of breast cancers with germline BRCA1 mutations and 23% with BRCA2 mutations fall into the category of TNBC, creating a therapeutic vulnerability exploitable through DNA damage response (DDR) targeting (Mavaddat et al., 2012). PARP inhibitors and platinum agents leverage this synthetic lethality by inducing DNA double-strand breaks that BRCA-deficient cells cannot repair. Although BRCA1/2 mutations increase tumor mutational burden, they paradoxically suppress host antitumor immunity (Kraya et al., 2019). By increasing cytoplasmic DNA concentration, PARP inhibitors activate the cGAS–STING pathway, promoting type I interferon production and enhancing T-cell infiltration into the previously immunosuppressed TME (Pantelidou et al., 2019). This dual action of direct tumor cell killing and TME immunomodulation facilitates overcoming resistance in TNBC. The differential expression mode of TROP2 in tumor and peripheral sites make it an ideal therapeutic intervention target in TNBC. In addition, TROP2 inhibitors are also empowered by advances in antibody drug conjugate techniques. The ASCENT clinical trial has established the role of sacituzumab govitecan (SG) in highly pretreated TNBC over chemotherapy with improved objective response rate (35% *versus* 5%) and median overall survival (12.7 months *versus* 6.7 months) (Bardia et al., 2021). The bystander effect refers to the cytotoxic impact of a leaked payload on neighboring cells. The superior efficacy of SG may be attributed to its prompt effects on the nearby microenvironment, which harbors numerous immunosuppressive cells. SG has a less stable linker compared to other ADC formulations, which facilitates bystander effects. SG provides a conceptual blueprint for drug design, where tumor-specific antigens (TSAs) such as TROP2 allow more precise targeting, and an easily cleavable linker disrupts the surrounding immunosuppressive TME. This strategy is particularly suited for highly malignant tumors such as TNBC, where a high mutational burden triggers robust antitumor immunity that is presumably rendered inactive by immunosuppression within the TME. Notably, some effector cytotoxic T cells may also be eliminated through bystander effects. Other ADC agents, such as anti-HER2 trastuzumab deruxtecan, offer new options for HER-2-low and HER2-ultra-low breast cancer patients, a considerable proportion of whom have historically been categorized as HER-2 negative patients, including a subset of TNBC patients (Denkert et al., 2021). HER2 is a prototypical oncoprotein with a differential expression mode between cancerous cells and TME cellularity. A growing number of bispecific antibodies are being developed for malignant contexts. Blinatumomab, a bispecific T-cell engager, exemplifies the common design of bispecific antibodies in malignancies. It simultaneously engages with CD19 on malignant B cells and CD3 on cytotoxic T cells to generate a killing effect. Blinatumomab exerts cytotoxic effects independently of antigen presentation by linking T cells directly to tumor cells, thereby presumably shortening the effector pathways and enhancing efficiency. Bispecific antibodies represent a promising avenue as they utilize antitumor cytotoxicity by binding tumor cells to T cells. In addition, TNBC tumors tend to have more frequent EGFR overexpression, which also motivates several preclinical explorations (Corkery et al., 2009).

Androgen receptor (AR) expression characterizes a distinct subtype of TNBC, the luminal androgen receptor (LAR) subtype, accounting for approximately 10%–15% of TNBC (Stella et al., 2023). AR signaling in TNBC orchestrates complex crosstalk within the TME. Preclinical studies demonstrate that enzalutamide, a second-generation AR antagonist, increases CD8^+^ T-cell infiltration in AR-positive TNBC tumor tissues and enhances the effectiveness of immune checkpoint inhibitors. A phase II trial (TBCRC 032) evaluating enzalutamide combined with taselisib in AR-positive TNBC reported a clinical benefit rate of 35.7% at 16 weeks. Paired tumor biopsies revealed treatment-induced modulation of the TME, including alterations in innate immune genes, adaptive immunity pathways, and mTOR signaling (Lehmann et al., 2020). Furthermore, the AR blockade could disrupt reciprocal signaling between cancer cells and CAFs. In prostate cancer, IL-6 from cancer cells activates AR signaling through STAT3, while CAFs produce growth factors (IGF, FGF, and TGF-β) that sustain AR activity (Culig et al., 2002). AR signaling in CAFs also suppresses pro-tumorigenic cytokines such as CCL2 and CXCL8, and AR inhibition increases their secretion, promoting cancer cell migration (Cioni et al., 2018). Similar mechanisms may operate in AR-positive TNBC. The combination of AR antagonists with immune checkpoint inhibitors has shown synergistic effects in preclinical models as the AR blockade combined with androgen deprivation therapy enhances CD8^+^ T-cell function and responsiveness to anti-PD-1 therapy by increasing IFN-γ expression and preventing T-cell exhaustion (Guan et al., 2022). Despite progress, challenges persist in developing predictive biomarkers beyond AR positivity. AR expression alone poorly predicts therapeutic response. The TME composition, AR transcriptional activity, and AR variant expression may offer better patient stratification (You et al., 2022).

The integration of targeted therapies into TNBC treatment paradigms introduces distinct toxicity profiles that influence patient compliance. For PARP inhibitor-based regimens, predominant adverse events encompass gastrointestinal reactions (e.g., nausea, diarrhea, vomiting, and anorexia) and hematologic toxicities. Among them, anemia is the sole adverse event exceeding 10% incidence at grade ≥3 severity (Zheng et al., 2024). The combination of PARP inhibitors and pembrolizumab in triple-negative breast cancer demonstrates promising efficacy. However, hematologic toxicities such as anemia and thrombocytopenia, along with immune-related adverse events, are commonly observed. Careful patient monitoring and dose adjustments are essential for optimal treatment outcomes. Antibody–drug conjugates, exemplified by SG and trastuzumab deruxtecan (T-DXd), introduce unique toxicity considerations. These toxicities stem from both the antibody component and the cytotoxic payload. The bystander effect enhances therapeutic efficacy by disrupting immunosuppressive cells within the tumor microenvironment. However, it simultaneously poses risks to normal tissue (Metrangolo et al., 2025; Staudacher and Brown, 2017). SG has a less stable linker design. Although this is advantageous for TME modulation, it contributes to higher rates of adverse events (Tolaney et al., 2025). These include neutropenia, diarrhea, and nausea compared to traditional chemotherapy. Grade 3–4 neutropenia occurs in approximately 51% of patients. Patient adherence is significantly influenced by both the burden of adverse events and the complexity of toxicity management.

## 12 Current global landscapes of TNBC clinical trials and implications in TME modulation

Optimizing TNBC management has long been a focus of research, with an array of approved regimens (Table 1). As of 2025, the clinical research for TNBC continues to examine diverse therapeutic modalities, including immune checkpoint inhibitors, ADCs and PARP inhibitors, and novel bispecific antibodies (Table 2). Recent phase III trials presented at the ESMO Congress 2025 have demonstrated the transformative efficacy of TROP2-targeted ADCs in the first-line metastatic setting. ASCENT-03 showed SG achieving a median PFS of 9.7 *versus* 6.9 months with chemotherapy (*p* < 0.001) (Cortes et al., 2025). TROPION-Breast02 demonstrated datopotamab deruxtecan (Dato-DXd) achieving a median PFS of 10.8 *versus* 5.6 months (*p* < 0.0001). AR antagonists have demonstrated modest clinical activity in the LAR subtype of TNBC, which accounts for 6.6%–75% of cases depending on AR expression thresholds. Enzalutamide achieved a clinical benefit rate of 33% at 16 weeks in patients with AR-positive TNBC expressing ≥10% nuclear AR (Traina et al., 2018). Recently, the phase II UCBG 3-06 START trial evaluating darolutamide reported an overall clinical benefit rate of 29% at 16 weeks but notably achieved 57% clinical benefit in molecular apocrine-high tumors compared to 16% in other tumor subtypes. In addition, darolutamide demonstrated a favorable tolerability with only 15% grade 3 or worse adverse events and no grade 4–5 events (Bonnefoi et al., 2025). Strategies combining AR inhibitors with CDK4/6 or PI3K inhibitors have also shown synergistic effects. PIK3CA mutations are significantly enriched in AR-positive TNBC (40% *versus* 4% in AR-negative cases) (Lehmann et al., 2014). The TBCRC 032 trial demonstrates a 75% clinical benefit rate for enzalutamide plus taselisib in LAR subtype patients (Lehmann et al., 2020). The PI3K pathway represents one of the most frequently dysregulated signaling cascades in TNBC. PIK3CA, AKT1, or inactivating PTEN mutation occurs in approximately 25%–30% of advanced TNBC cases (Pascual and Turner, 2019). Eganelisib is a first-in-class, highly selective PI3K-γ inhibitor. The MARIO-3 trial investigating eganelisib combined with atezolizumab and nab-paclitaxel showed promising activity in first-line metastatic TNBC. The 1-year PFS rate was 36.0% compared to 23.7% in the IMpassion130 benchmark trial (O'Connell et al., 2024). Notably, clinical benefit was observed irrespective of PD-L1 expression status. Typically, PD-L1-negative TNBC exhibits poor response to checkpoint inhibitor monotherapy (Adams et al., 2019b). Mechanistically, eganelisib overcomes this resistance by reprogramming TAMs from the M2 phenotype to the M1 phenotype. It might be attributed to the role of PI3K-γ in the maintenance of M2 TAMs. In contrast, ipatasertib, a pan-AKT inhibitor, did not improve the efficacy of atezolizumab plus paclitaxel in patients with PD-L1-positive TNBC in the phase III IPATunity170 trial (Schmid et al., 2024). This underscores a distinction between targeting downstream effectors such as AKT *versus* selectively inhibiting PI3K-γ in the context of immuno-oncology combinations. Unlike AKT, PI3K-γ is predominantly expressed in myeloid cells, which largely play immunosuppressive roles. The different outcomes highlight the importance of accurately modulating the TME.)

**TABLE 1:** Clinical trials with approved agents in TNBC.

Drug name	Phase	Drug class	Target	Key trial(s)	Status	Setting
Sacituzumab govitecan (Trodelvy®)	Phase III	ADC	TROP-2	ASCENT	FDA-approved 2020 (2L+)	≥2 prior therapies for metastatic TNBC
Trastuzumab deruxtecan (Enhertu®)	Phase III	ADC	HER2	DESTINY-Breast04	FDA-approved 2022	HER2-low metastatic BC (including TNBC)
Pembrolizumab (Keytruda®)	Phase III	ICI	PD-1	KEYNOTE-522	FDA-approved 2021	Neoadjuvant/adjuvant high-risk early TNBC
Pembrolizumab + chemotherapy	Phase III	ICI + chemo	PD-1	KEYNOTE-355	FDA-approved 2020	1L metastatic TNBC (PD-L1 CPS ≥10)
Olaparib (Lynparza®)	Phase III	PARP inhibitor	PARP	OlympiAD	FDA-approved 2018	gBRCA+ metastatic TNBC
Talazoparib (Talzenna®)	Phase III	PARP inhibitor	PARP	EMBRACA	FDA-approved 2018	gBRCA+ metastatic TNBC
Eribulin (Halaven®)	Phase III	Chemotherapy	Microtubules	EMBRACE	FDA-approved 2010	≥2 prior regimens for metastatic BC
Paclitaxel	Standard	Chemotherapy	Microtubules	Multiple trials	Standard of care	Various TNBC settings
Nab-paclitaxel (Abraxane®)	Phase III	Chemotherapy	Microtubules	—	FDA-approved 2005	Metastatic BC after failure of combo therapy
Carboplatin	Standard	Chemotherapy	DNA crosslinker	Multiple trials	Standard of care	Neoadjuvant and metastatic TNBC
Capecitabine (Xeloda®)	Phase III	Chemotherapy	Thymidylate synthase	—	FDA-approved 1998	Metastatic BC after anthracycline/taxane
Gemcitabine (Gemzar®)	Phase III	Chemotherapy	Nucleoside analog	—	FDA-approved 2004	Metastatic BC (combo with paclitaxel)
Doxorubicin	Standard	Chemotherapy	Anthracycline	Multiple trials	Standard of care	Neoadjuvant and adjuvant TNBC
Epirubicin	Standard	Chemotherapy	Anthracycline	Multiple trials	Standard of care	Neoadjuvant and adjuvant TNBC
Cyclophosphamide (Cytoxan®)	Standard	Chemotherapy	Alkylating agent	Multiple trials	Standard of care	Neoadjuvant and adjuvant TNBC (combo)
Atezolizumab + nab-paclitaxel (Tecentriq®)	Phase III	ICI + chemo	PD-L1	IMpassion130	Withdrawn 2021	1L metastatic TNBC (PD-L1+) - historic

Abbreviations: 1L, first-line; 2L+, second-line and beyond; ADC, antibody–drug conjugate; BC, breast cancer; Chemo, chemotherapy; CPS, combined positive score; DNA, deoxyribonucleic acid; FDA, Food and Drug Administration; gBRCA+, germline breast cancer susceptibility gene positive; HER2, human epidermal growth factor receptor 2; ICI, immune checkpoint inhibitor; PARP, poly (ADP-ribose) polymerase; PD-1, programmed cell death protein 1; PD-L1, programmed death-ligand 1; TNBC, triple-negative breast cancer; and TROP-2, trophoblast cell surface antigen 2.

**TABLE 2:** Ongoing clinical trials of TNBC.

Drug name	Phase	Drug class	Target	Key trial(s)	Status	Setting
Datopotamab deruxtecan	Phase III	ADC	TROP-2	TROPION-Breast03	Ongoing	Stage I–III TNBC with residual disease post-neoadjuvant
Datopotamab deruxtecan + durvalumab	Phase III	ADC + ICI	TROP-2 + PD-L1	TROPION-Breast04	Ongoing	Stage II–III TNBC or HR-low, HER2-low BC
Datopotamab deruxtecan ± durvalumab	Phase III	ADC ± ICI	TROP-2 ± PD-L1	TROPION-Breast05	Ongoing	PD-L1+ metastatic TNBC
Sacituzumab govitecan + pembrolizumab	Phase III	ADC + ICI	TROP-2 + PD-1	ASCENT-04, TroFuse-011	Ongoing	1L PD-L1+ metastatic TNBC
Sacituzumab tirumotecan ± pembrolizumab	Phase I/II	ADC ± ICI	TROP-2 ± PD-1	—	Ongoing	Advanced TNBC
Pembrolizumab vs. observation	Phase III	ICI	PD-1	OptimICE-PCR	Ongoing	Early TNBC with pCR after neoadj. chemo + pembrolizumab
Capivasertib + paclitaxel	Phase III	AKT inhibitor + chemo	AKT	CAPItello-290	Ongoing	1L advanced TNBC (all-comer population)
Ipatasertib + paclitaxel + atezolizumab	Phase III	AKT inhibitor + chemo + ICI	AKT + PD-L1	IPATunity130 cohort C	Ongoing	PIK3CA/AKT/PTEN non-altered metastatic TNBC
Bicalutamide + palbociclib	Phase II	Anti-androgen + CDK4/6i	AR + CDK4/6	NCT02605486	Ongoing	AR+ RB+ metastatic TNBC
Bicalutamide + ribociclib	Phase II	Anti-androgen + CDK4/6i	AR + CDK4/6	NCT03090165	Ongoing	AR+ metastatic TNBC
Enzalutamide + taselisib	Phase Ib/II	Anti-androgen + PI3Ki	AR + PI3K	NCT02457910	Ongoing	AR+ advanced TNBC
Enzalutamide + BYL719	Phase II	Anti-androgen + PI3Ki	AR + PI3K	NCT03207529	Ongoing	AR+ PTEN+ metastatic BC
Abiraterone + AZD8186	Phase I	CYP17i + PI3Ki	Androgen synthesis + PI3K	NCT01884285	Ongoing	Advanced TNBC
Adavosertib (AZD1775)	Phase II	WEE1 inhibitor	WEE1	Multiple trials	Ongoing	Advanced solid tumors including TNBC
Ceralasertib (AZD6738)	Phase I/II	ATR inhibitor	ATR	—	Ongoing	Advanced solid tumors including TNBC
Berzosertib (M6620)	Phase I/II	ATR inhibitor	ATR	—	Ongoing	Advanced solid tumors with chemo
KN046 + nab-paclitaxel	Phase II	PD-L1/CTLA-4 BsAb + chemo	PD-L1 + CTLA-4	NCT03872791	Ongoing	1L metastatic TNBC
Adagloxad simolenin (OBI-822/OBI-821)	Phase III	Cancer vaccine	Globo-H	GLORIA	Ongoing	Adjuvant high-risk, early stage Globo-H+ TNBC
A2B395 (tmod CAR T)	Phase I/II	CAR T-cell	EGFR (HLA-A*02 loss)	—	Ongoing	EGFR+ HLA-A*02 loss solid tumors including TNBC
CRX100 ± pembrolizumab	Phase I	Adoptive cell therapy	Personalized	—	Ongoing	Advanced solid malignancies including TNBC
Cediranib + olaparib	Phase II	VEGFRi + PARPi	VEGFR + PARP	—	Ongoing	Advanced/metastatic unresectable TNBC

Abbreviations: 1L, first-line; ADC, antibody–drug conjugate; AKT, protein kinase B; AR, androgen receptor; ATR, ataxia telangiectasia and Rad3-related; BC, breast cancer; BsAb, bispecific antibody; CAR T, chimeric antigen receptor T cell; CDK4/6i, cyclin-dependent kinase 4/6 inhibitor; Chemo, chemotherapy; CTLA-4, cytotoxic T-lymphocyte-associated protein 4; CYP17i, cytochrome P450 17A1 inhibitor; DDR, DNA damage response; EGFR, epidermal growth factor receptor; HER2, human epidermal growth factor receptor 2; HLA, human leukocyte antigen; HR-low, hormone receptor-low; ICI, immune checkpoint inhibitor; PARPi, poly (ADP-ribose) polymerase inhibitor; pCR, pathologic complete response; PD-1, programmed cell death protein 1; PD-L1, programmed death-ligand 1; PI3K, phosphatidylinositol 3-kinase; PI3Ki, phosphatidylinositol 3-kinase inhibitor; PTEN, phosphatase and tensin homolog; RB, retinoblastoma; TNBC, triple-negative breast cancer; TROP-2, trophoblast cell surface antigen 2; VEGFR, vascular endothelial growth factor receptor; VEGFRi, vascular endothelial growth factor receptor inhibitor; and WEE1, WEE1 kinase.

PARP inhibitors represent the first targeted therapy approved in TNBC. The candidates for PARP inhibitor therapy constitute a group of TNBC patients who harbor germline BRCA1/2 mutations (Kast et al., 2016). The phase III OlympiAD trial established olaparib as superior to single-agent chemotherapy in patients with germline BRCA-mutated, HER2-negative metastatic breast cancer. Similarly, the phase III EMBRACA trial demonstrated better efficacy of talazoparib over chemotherapy in the same patient population (Litton et al., 2018). More recently, the OlympiA trial evaluated adjuvant olaparib in patients with high-risk, early-stage, HER2-negative breast cancer harboring germline BRCA1/2 mutations. The trial demonstrated that adjuvant olaparib significantly improved invasive DFS (3-year invasive DFS: 85.9% vs. 77.1%) (Tutt et al., 2021). These results established PARP inhibition as a viable strategy for BRCA-mutated TNBC. Combination strategies face challenges, including overlapping hematological toxicities and the potential for antagonistic interactions with DNA-damaging agents. Platinum-based chemotherapy also plays a role in TNBC management, particularly in the neoadjuvant setting. The addition of platinum agents to standard anthracycline- and taxane-based regimens improves pCR rates. A meta-analysis of 11 randomized controlled trials demonstrated that platinum-containing regimens achieved pCR rates of 40% compared to 27% with non-platinum regimens. This improvement comes at the cost of increased hematological toxicities, including neutropenia and thrombocytopenia. Despite encouraging results, the optimal patient selection criteria for platinum-based therapy remain incompletely defined. Molecular subtyping of TNBC based on TME cellular composition may help refine patient selection in this regard. Bispecific antibodies represent an emerging class of immunotherapeutics that simultaneously engage tumor-associated antigens and immune effector cells. HER2 × CD3 bispecific antibody-armed activated T cells (HER2 BATs) have demonstrated encouraging results in both high-level and low-level HER2-expressing breast cancers (Lum et al., 2021). This trial of HER2 BATs as consolidation therapy reported a median overall survival of 13.1 months, with evidence of sustained immune activation. Dual-immune checkpoint blockade bispecific antibodies are also under investigation. A phase II study evaluated KN046, a PD-L1 and CTLA-4 bispecific antibody, combined with nab-paclitaxel in first-line metastatic TNBC. The combination achieved an ORR of 44% and a median PFS of 7.3 months, with greater benefit in PD-L1-positive patients (Li et al., 2024). Novel bispecific constructs targeting TROP-2 × CD3 are in preclinical development. TROP-2 is highly expressed in the majority of TNBC tumors but minimally expressed in normal tissues. Bispecific T-cell engagers aim to redirect cytotoxic T cells to TROP-2-expressing tumor cells. Preclinical studies using three-dimensional MDA-MB-231 spheroid models have demonstrated potent antitumor activity of them (Liu Y. et al., 2022). Taken together, bispecific antibodies for TNBC warrant further investigation. Given that bispecific T-cell engagers function by redirecting and activating cytotoxic T cells against tumor cells, combining these agents with strategies that program the immunosuppressive TME may generate more favorable effects. For example, it could be combined with agents suppressing immunosuppressive cell populations within the TME, including Tregs, MDSCs, and TAMs. Moreover, neutralizing immunosuppressive cytokines such as TGF-β and IL-10 could also potentially alleviate the immunosuppressive TME.

## 13 Current status and future implications

Emerging research is redefining the TNBC tumor TME as not merely a passive scaffold but a complementary part. Recent advances have revealed how TME networks drive immune evasion and treatment resistance (Hegde and Chen, 2020). Consequently, identifying TME modulators and their therapeutic vulnerabilities showed promising clinical relevance (Valkenburg et al., 2018). Specifically, key players include CAFs, TAMs, diverse immune cell populations, and metabolic intermediates that collectively shape therapeutic outcomes. Understanding the molecular mechanisms governing TME dynamics represents a critical frontier for effective interventions in TNBC. The mechanisms controlling stromal cell plasticity within the tumor niche are poorly characterized. TAMs, for instance, display remarkable functional versatility, yet the deterministic signals underlying their pro- *versus* antitumor phenotypes require further clarification. Similarly, the precise mechanisms by which regulatory T cells modulate immune responses across different TNBC subtypes remain to be elucidated. Current immunotherapy approaches targeting PD-1/PD-L1 benefit only a small fraction of patients (Adams et al., 2019c). Dissecting immune cell mechanisms within the TME and their reprogramming potential may enable subtype-specific treatment selection and optimize combinatorial strategies (Liu K. et al., 2022). Advanced technologies are accelerating these efforts. Spatial transcriptomics enables mapping of cellular interactions within their native tissue architecture (Hunter et al., 2021). Single-cell profiling reveals functional heterogeneity among immune populations (Papalexi and Satija, 2018). Multi-omics integration further captures dynamic state transitions during TNBC treatment (Lehmann et al., 2021). In addition, artificial intelligence (AI) has become integral to pathology practice and research, with computational pathology now serving as a paradigm for AI-driven medicine (Aggarwal et al., 2025). Deep learning algorithms can quantify tumor-infiltrating lymphocytes with accuracy matching expert pathologists, providing objective immune scoring systems (Zhang H. et al., 2025). AI-powered image analysis identifies spatial patterns of immune cell distribution that correlate with clinical outcomes, such as the distance between CD8^+^ T cells and tumor nests or the density of tertiary lymphoid structures (van Rijthoven et al., 2024). AI also assists in identifying novel TME biomarkers by analyzing thousands of cellular features imperceptible to human observers, including subtle morphological changes in stromal cells (Xie et al., 2024). These computational tools not only accelerate TME characterization but also enable real-time TME dynamic monitoring through serial biopsy analysis during treatment. All these approaches collectively provide unprecedented resolution to decode TME complexity and identify actionable targets.

Current therapeutic modalities are beginning to modulate the TNBC TME, either intentionally or inadvertently (Zhang B. et al., 2025). Generally, chemotherapeutic agents and PARP inhibitors increase tumor immunogenicity by enhancing neoantigen generation (Humer et al., 2025). In addition, low-dose chemotherapy administered in a metronomic fashion preferentially depletes immunosuppressive myeloid cells while sparing effector lymphocytes (Tagliamonte et al., 2016). Similarly, the timing of immunotherapy administration to enhance chemotherapy-induced immune activation is critical for maximizing synergistic effects (Zhang B. et al., 2025). Modest modifications to existing regimens that deliberately target TME vulnerabilities may thus achieve clinical gains without requiring entirely novel drug development.

## 14 Conclusion

Despite recent therapeutic advances, TNBC remains a clinical challenge requiring innovative treatment strategies. Current evidence indicates that targeting the TME represents a promising therapeutic avenue that warrants further investigation. Recent single-cell and spatial transcriptomic analyses have revealed the complex cellular heterogeneity within the TNBC TME. They have facilitated research on multiple immunosuppressive cell populations, including CAFs, TAMs, and TANs. These stromal cells demonstrate diverse functional states and engage in complex crosstalk that collectively sustains immunosuppression and promotes tumor progression. Targeting specific cell populations has shown limited efficacy due to compensatory mechanisms from other immunosuppressive components. Therefore, future therapeutic strategies should focus on (i) simultaneously targeting multiple stromal cell types, for example, CAFs and TAMs, to prevent compensatory immunosuppression; (ii) disruption of shared metabolic reprogramming pathways across different TME cell populations, such as glycolysis and lipid metabolism, to deprive immunosuppressive cells of energy resources; (iii) normalization of tumor vasculature to enhance immune cell trafficking and penetration; (iv) inhibition of critical signaling pathways mediating stromal-immune cell crosstalk, such as TGF-β and CXCL12-CXCR4 signaling; and (v) development of sequential combination approaches that integrate TME-remodeling agents with immune checkpoint inhibitors and conventional cytotoxic therapies to achieve synergistic antitumor effects. These multi-modal strategies may overcome the redundancy in immunosuppressive mechanisms and improve therapeutic outcomes for TNBC patients.
